# Circular RNA atlas in osteoclast differentiation with and without alendronate treatment

**DOI:** 10.1186/s13018-020-01722-6

**Published:** 2020-07-01

**Authors:** Jianbiao Lin, Shaofeng Ma, Cong Zhu, Changqing Chen, Weibin Lin, Canbin Lin, Guofeng Huang, Zhenqi Ding

**Affiliations:** 1 Center for Orthopedics, Affiliated Southeast Hospital of Xiamen University/909th Hospital of People’s Liberation Army, 269 Zhanghua Middle Road, Zhangzhou, 363000 Fujian China; 2 Obstetrics and Gynecology Department, Affiliated Southeast Hospital of Xiamen University/909th Hospital of People’s Liberation Army, Zhangzhou, China

**Keywords:** Alendronate, Osteoporosis, MCSF, RANKL

## Abstract

**Background:**

Alendronate (AL) is the most widely used bisphosphonate in the treatment of osteoporosis (OP). However, the role of circular RNAs (circRNAs) in the treatment of OP with AL remains unclear.

**Methods:**

In this study, we showed that osteoclast (OC) precursors (OPCSs) could be induced into OCs with macrophage colony-stimulating factor (MCSF) and receptor activator of nuclear factor-κB ligand (RANKL) treatment. Subsequently, the OCs were treated with AL. OC differentiation-related biomarkers including RANK, tartrate-resistant acid phosphatase (TRAP), and cathepsin K (CTSK) were analyzed with TRAP staining, quantitative real-time (qPCR), and western blotting. Differentially expressed circRNAs (DECs) were identified among the OPCS, OC, and OC + AL groups. In addition, the expression levels of 10 DECs related to OC differentiation were verified by qPCR.

**Results:**

TRAP staining showed that MCSF and RANKL treatment effectively induced OPCSs to differentiate into OCs. In addition, qPCR and western blot analysis revealed that the three biomarkers of OC (RANK, TRAP, and CTSK) were expressed significantly more in the OC group than those in the OPCS group. In contrast, the mRNA and protein expression levels of these three biomarkers decreased significantly in OCs treated with AL compared with those non-treated OCs. GO analysis of the DECs in the OPCS group vs. the OC group revealed that their functions were mainly related to cell, cell part, binding, and single-organism terms. KEGG analysis of the top 20 DECs in a comparison between the OPCS and OC groups showed that genes involved in mitogen-activated protein kinase signaling were the most common. Results of functional analyses of DECs in an OC vs. OC + AL comparison were similar to those in the OPCS vs. OC comparison. Finally, qPCR showed that, in the OC + AL vs. OC group comparison, the expression levels of seven and three DECs significantly decreased and increased, respectively.

**Conclusions:**

Having successfully induced OPCSs to differentiate into OCs, we showed that AL suppresses the differentiation of OPCS into OC and that 10 DECs were involved in the regulation of this process. This indicates that these DECs might be important to the treatment of OP.

## Introduction

Osteoporosis (OP) is among the most common bone diseases worldwide. It is characterized by a reduction in bone tissue volume and bone density per unit volume, and it is caused by the loss of bone calcium and bone matrix [1]. Because the average age of populations is generally increasing, OP is becoming a worldwide health problem. Indeed, according to the International Osteoporosis Foundation, more than 200 million people worldwide have OP, with the most common type being postmenopausal OP [2] (which about 30% of postmenopausal women suffer from). The most important mechanisms of postmenopausal OP are the excessive activation of osteoclasts (OCs) and the inhibition of osteoblasts caused by estrogen deficiency. Moreover, attenuation of osteoblast formation and/or increased bone resorption by OCs is a key mechanism of pathogenesis in OP; thus, OC dysfunction is an important OP pathogenic factor [3, 4]. Although previous studies have revealed multiple molecular mechanisms for OC dysfunction, few have investigated the roles of circular RNAs (circRNAs) during drug-related regulation of OC differentiation.

Osteoblasts and OCs are the main functional cells of bone formation: osteoblasts are responsible for new bone formation, whereas OCs are responsible for aged bone resorption [5]. Thus, these cells maintain a dynamic balance of formation and resorption; however, when this balance is disrupted, the function or architecture of the bone becomes abnormal [6]. Consequently, drugs for the treatment of OP mainly include bone resorption inhibitors and bone formation promoters. One such drug is alendronate (AL), which is a bisphosphonate widely used for OP treatment. AL has multiple effects in OP patients including inhibiting OC activity, increasing bone density, and reducing the incidence of vertebral and non-vertebral fractures [7, 8]. Previous studies have suggested that bisphosphonate's mechanism of action is dependent on three processes: (1) altering the morphology and structure of the OCs to inhibit their function; (2) inhibiting the production of osteoblast-mediated interleukin (IL)-6 and tumor necrosis factor-α, thereby decreasing OC genesis; and (3) physically and chemically bonding with the bone matrix, thereby interfering with bone resorption [9]. However, the role of circRNAs in the underlying molecular mechanisms of AL’s effects in OP remains to be elucidated.

circRNAs are covalently closed RNA molecules that are generated through a process named back-splicing [10] and are involved in the regulation of various biological processes. For example, circRNA.014511 inhibits P53 expression by binding to miR-29b-2-5p, and it reduces the sensitivity of bone marrow mesenchymal stem cells by regulating cell apoptosis and the cell cycle [11]. Additionally, circRNA.33186-knockdown inhibits apoptosis and promotes proliferation in chondrocytes treated with IL-1β [12]. Also, circRNA.28313 functions in macrophage colony-stimulating factor (MCSF) + receptor activator of nuclear factor-κB ligand (RANKL)-induced OC differentiation via the regulation of miR-195a expression; this affects bone absorption in mice [13]. Moreover, at different stages of mouse OC growth, the expression levels of various circRNAs are different [14].

Unfortunately, OCs are difficult to separate from cortical bone in vitro: they are terminally differentiated cells lacking proliferation abilities [15]. Therefore, OCs cannot be isolated for culture in vitro. However, THP-1 cells can differentiate into OCs when stimulated by phorbol-12 myristate-13 acetate (PMA), RANKL, and MCSF [16, 17]. Thus, to investigate the involvement of circRNAs in the AL treatment of OP, THP-1 cells were used in the present study to differentiate into OCs for further functional analysis. Specifically, we probed the potential circRNA atlas involved in AL-induced OC differentiation. From our results, we were able to draw a circRNA–miRNA–mRNA network of AL-induced OC differentiation. Our results provide insights that could be used for further clinical studies of OP treatment.

## Materials and methods

### Cell treatment

THP-1 cells were purchased from ATCC (VA, USA) and maintained in RPMI 1640 with 10% fetal bovine serum (Invitrogen, Carlsbad, CA, USA) and 1% penicillin/streptomycin (HyClone, USA). Cell lines were maintained in a humidified chamber at 5% CO_2_ and 37 °C. THP-1 cells were induced into OC precursors (OPCSs) through stimulation with 100 ng/mL PMA for 3 days. The detailed experimental procedure for this process was previously described by Takashiba et al. [18]. Once OPCSs were available, they were uniformly inoculated into 96-well plates (5000 cells/well); they were induced over 7 days into OCs using a final concentration that contained 100 ng/mL RANKL and 50 ng/mL MCSF. Every 72 h during induction, the culture medium was changed and the state of OC differentiation was evaluated. During OC differentiation, 10^−8^ M AL was either added or not added (depending on the treatment group) to the induction culture on day 4. After 7 days, the treated cells were harvested and divided into three groups: the OPCS group, OC group, and OC + AL group.

### Tartrate-resistant acid phosphatase (TRAP) staining

Here, TRAP staining was performed by modifying the method of Oshima et al. [19]. Briefly, OCs were incubated for 15 min at 37 °C in freshly prepared 0.1 M Tris buffer (pH 5.0) that contained 1.35 mM naphthol AS-MX phosphate (Sigma); 0.362 M *N*, *N*-dimethylformamide; 3.88 mM Violet LB salt (Sigma); and 25 mM sodium tartrate. Slides were rinsed for 10 min and counterstained with hematoxylin. One-micromolar sections were cut using a Sorvall Porter-blum MT-2B ultra microtome. New bone formation was visualized using fluorescence microscopy (Nikon, Tokyo, Japan).

### Real-time quantitative PCR (qPCR)

Reverse transcription of mRNA from the three treatment groups was carried out using a final volume of 100 μL from 400 ng total RNA and with a High-capacity cDNA Archive kit (Applied Biosystems) according to the manufacturer’s instructions. mRNA levels were determined by qPCR, and the following primers were used: RANK forward 5′-TCTGCTTCTCTTCGCGTCTG-3′; RANK reverse 5′-AGCCTCATTGATCCAGTGCC-3′; TRAP forward 5′-GATCCCACAGACCAATGTGTC-3′; TRAP reverse 5′-CCAGCACGTAGTCCTCCCT-3′; cathepsin K (CTSK) forward 5′-TCCTGTTGGGCTTTTAGCTC-3′; CTSK reverse 5′-GTCATGTAGCCCCCTCCAC-3. Reactions were performed in 50-μL volumes containing SYBR Green PCR master mix (Perkin-Elmer Biosystems). qPCR was performed using 96-well optical plates in a GeneAmp PCR System 9600 (Perkin-Elmer Biosystems). Thermal cycling conditions were as follows: 2 min at 50 °C and 10 min at 95 °C, followed by 40 cycles at 95 °C for 30 s, 60 °C for 30 s, and 72 °C for 2 min. Data were collected using an ABI analytical thermal cycler. mRNA expression levels were calculated on the basis of a relative standard curve and using the 2^−ΔΔct^ method. In addition, we designed 20 primer pairs to identify the linear DNA and corresponding circRNAs; the primer sequences for these are listed in Tables 1 and 2. PCR products were analyzed using agarose gel electrophoresis (1.5%).

**Table 1:** Primer sequences of circRNAs

CircBase ID	Sequence (5′ to 3′)	Product length	Gene ID
hsa_circ_0000284	TCTCGGTACTACAGGTATGGC	193	HIPK3
	ACCCTTAGTGGGAGGATGAGA		
hsa_circ_0000638	CGGGAAAAATAGTAGCACCAGC	173	ETFA
	TGCCACCTTGTCACATTTGG		
hsa_circ_0000994	TGAAATTGTTAGGTTGTGACAGTTG	158	SLC8A1
	TCTCCTTCCATTTCTGTCTCAGC		
hsa_circ_0001776	CAAGGAACCTTCCGGGTGTT	143	ESYT2
	GCTTTGGAAGATTTGGTTGCCA		
hsa_circ_0002922	TGAGGCAAGACTTCTAACTCGG	165	ZNF124
	GCTCTGGTCTTCCCCTTTGT		
hsa_circ_0003249	AATCATTCCTGTGATGCCAGA	186	LRP11
	TGAGGCACCTTCATGTCCAC		
hsa_circ_0007710	CCAGAAGGAAGACAATGCTGTT	173	ELF2
	AGTCAATAGAGATGGAGTGGAGT		
hsa_circ_0094798	ATGAGAATGGCATCTAAGAATGAAG	163	CWF19L2
	CCCTTTCTCTTTCCTCATGGGT		
hsa_circ_0101874	GGACAGCCTGATGCCAAAAC	200	FKBP3
	CAGACTTGGTTTCTTTGGGTTTA		
hsa_circ_0113954	ACAAAGAAAATGAAAAAGGAAGGCG	173	MIER1
	TCCCCACTACAGCCACTGTT

**Table 2:** Primer sequences of qPCR

Gene	Primer	Sequence (5′ to 3′)	Product length (bp)
HIPK3	H-HIPK3-F	CAGTCTTCCTTCTCCGCTCC	166
	H-HIPK3-F	CTTCCTTCCCGGGGATTTGG	
ETFA	H-ETFA-F	CTCCACCAGCCTAATCAACCT	196
	H-ETFA-F	TGGAGCTGGGTCAGGTTTAAG	
SLC8A1	H-SLC8A1-F	CATCGAAGGGACTGCCAGAG	181
	H-SLC8A1-F	CTCACTCATCTCCACCAGGC	
ESYT2	H-ESYT2-F	AAGGAACCTTCCGTCAGGG	120
	H-ESYT2-F	TCCCACACAGGTTCATTGGT	
ZNF124	H-ZNF124-F	CTTCTCCTTCACTCTCGGCG	180
	H-ZNF124-F	TCCAACAAAGCCCACTCCTC	
LRP11	H-LRP11-F	TGAGTCAAAGGGTGATGGAGG	121
	H-LRP11-F	TAGTCGGCATGCAACCATGA	
ELF2	H-ELF2-F	TCCTTCTCTCCCTGTGGAGC	151
	H-ELF2-F	TCTTTGGAACTGCTCTCCGC	
CWF19L2	H-CWF19L2-F	TGGTGGATTAAGCTGGCTAAGG	195
	H-CWF19L2-F	TATGTGGGTTTCCTCCACCG	
FKBP3	H-FKBP3-F	AGTAAAGCGGAGGCAGCG	191
	H-FKBP3-F	TGTTAGCTGTCTTGGCCACAT	
MIER1	H-MIER1-F	TGAATCTTCAAGTCCAGGAGGT	182
	H-MIER1-F	GAATTGGCATGTCGCCTTCC	

### Western blot analysis

Total cellular protein from the three treatment groups was isolated by the addition of 1% PMSF and RIPA lysis buffer (containing 50 mM Tris–HCl at pH 7.4, 150 mM NaCl, 1% NP-40, and 0.1% SDS). After boiling with SDS-PAGE sample buffer for 5 min, the samples were subjected to SDS-PAGE. The proteins were then transferred to a PVDF membrane (Millipore, USA). After blocking for 1 h at room temperature, the membrane was incubated with a 1:1000 dilution of rabbit polyclonal anti-mouse RANK, TRAP, CTSK, or GAPDH antibodies (ABGENT, USA) overnight. Before detection with an enhanced chemiluminescence detection kit (Advansta, USA), proteins were incubated with the corresponding secondary antibody for 1 h at room temperature. The bands were obtained by GeneGnome 5 (Synoptics Ltd., UK).

### Bioinformatics

Differentially expressed circRNAs (DECs) were determined using the following parameters: *P* value ≤ 0.05 and |Log_2_Ratio| ≥ 2. GO enrichment analysis and KEGG analysis of DECs were carried out using the online database KOBAS3.0 (http://kobas.cbi.pku.edu.cn/) with the default parameters maintained. In addition, a hypergeometric test was used to calculate the major biochemical metabolic pathways in which the DECs were involved.

### Data analysis

SPSS V16.0 software (IBM, USA) was used for statistical analyses. Statistical differences between groups were calculated using Tukey’s test. Where *P* was < 0.05, this indicated a significant difference.

## Results

### OC differentiation and drug induction

**Fig. 1:**
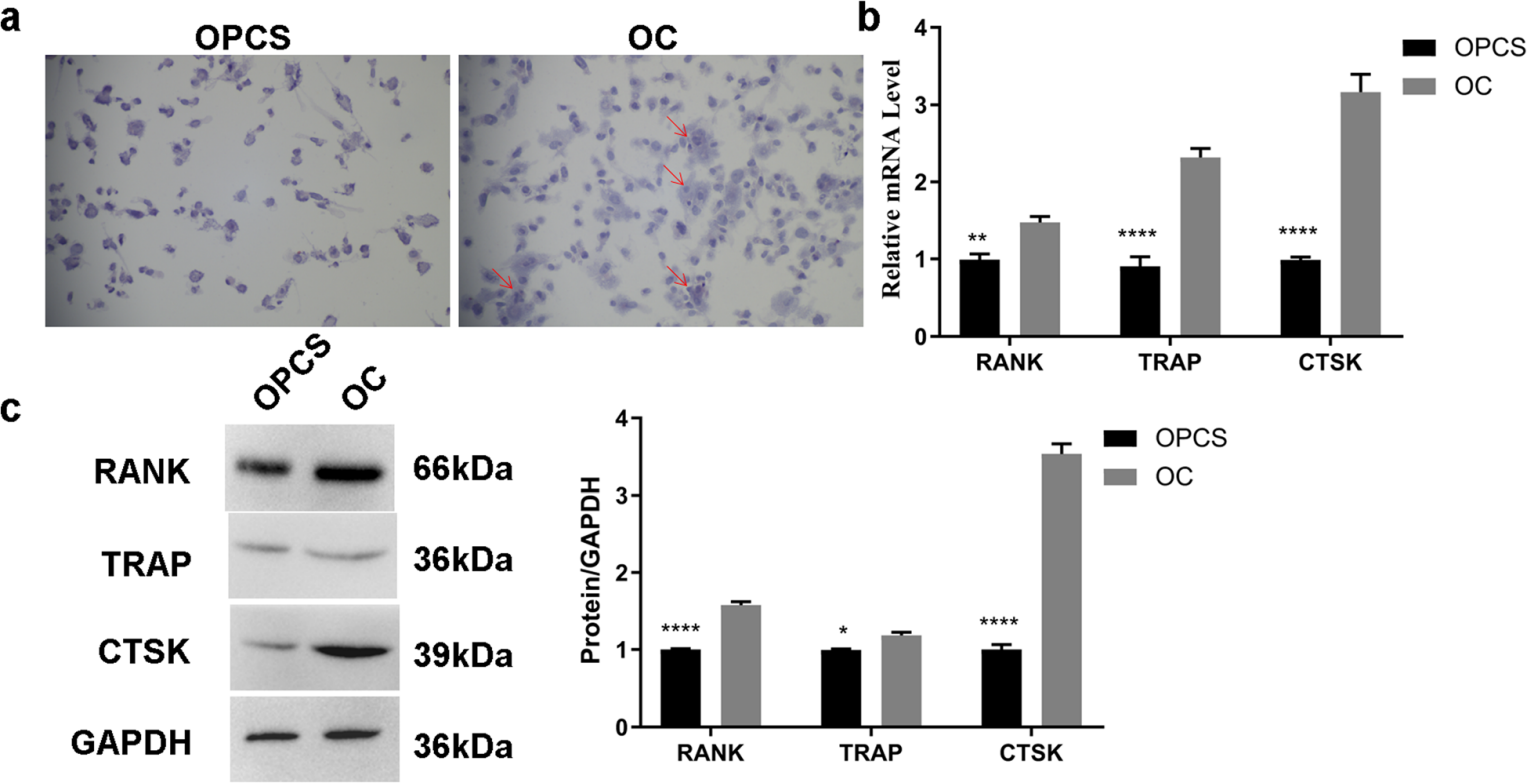
Analysis of OPCS differentiation into OCs. **a** Morphological examination of OPCS differentiation into OCs with TRAP staining. **b** mRNA abundance of OC differentiation-related genes in OPCSs and OCs. **c** Western blot analysis of OC differentiation-related proteins in OPCSs and OCs. *P < 0.05, **P < 0.01, and ****P < 0.0001. GAPDH was used as an internal reference. OCs, osteoclasts; OPCSs, osteogenic precursor cells

**Fig. 2:**
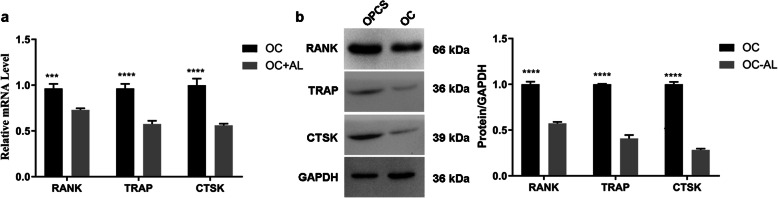
Analysis of AL-treated OCs. **a** mRNA abundance of OC differentiation-related genes in AL-treated OCs and non-treated OCs. **b** Western blot analysis of OC differentiation-related genes in AL-treated OCs and non-treated OC cells. ***P < 0.001 and ****P < 0.0001. GAPDH was used as an internal reference. AL, alendronate; OCs, osteoclasts

In order to investigate the OC differentiation and AL induction of OCs, THP-1 cells were induced into OPCSs by PMA treatment. Subsequently, OPCSs were further induced into OCs by MCSF and RANKL treatment. Finally, AL was used to treat OC. TRAP staining showed that MCSF and RANKL treatment effectively induced OPCS to differentiate into OCs (Fig. 1a). Moreover, qPCR showed that the expression levels of the OC differentiation-related biomarkers RANK, TRAP, and CTSK were significantly increased in OCs compared with OPCSs (*P* < 0.05) (Fig. 1b). Western blot analysis confirmed these results (*P* < 0.05) (Fig. 1c). In the AL induction of OCs, qPCR showed that the expression levels of RANK, TRAP, and CTSK were significantly decreased in AL-treated OCs compared with those in untreated OCs (*P* < 0.05) (Fig. 2a). Similar results were observed in western blot analysis (Fig. 2b). Thus, AL apparently inhibited the differentiation of OCs.

### DECs in an OPCS group vs. OC group comparison

**Fig. 3:**
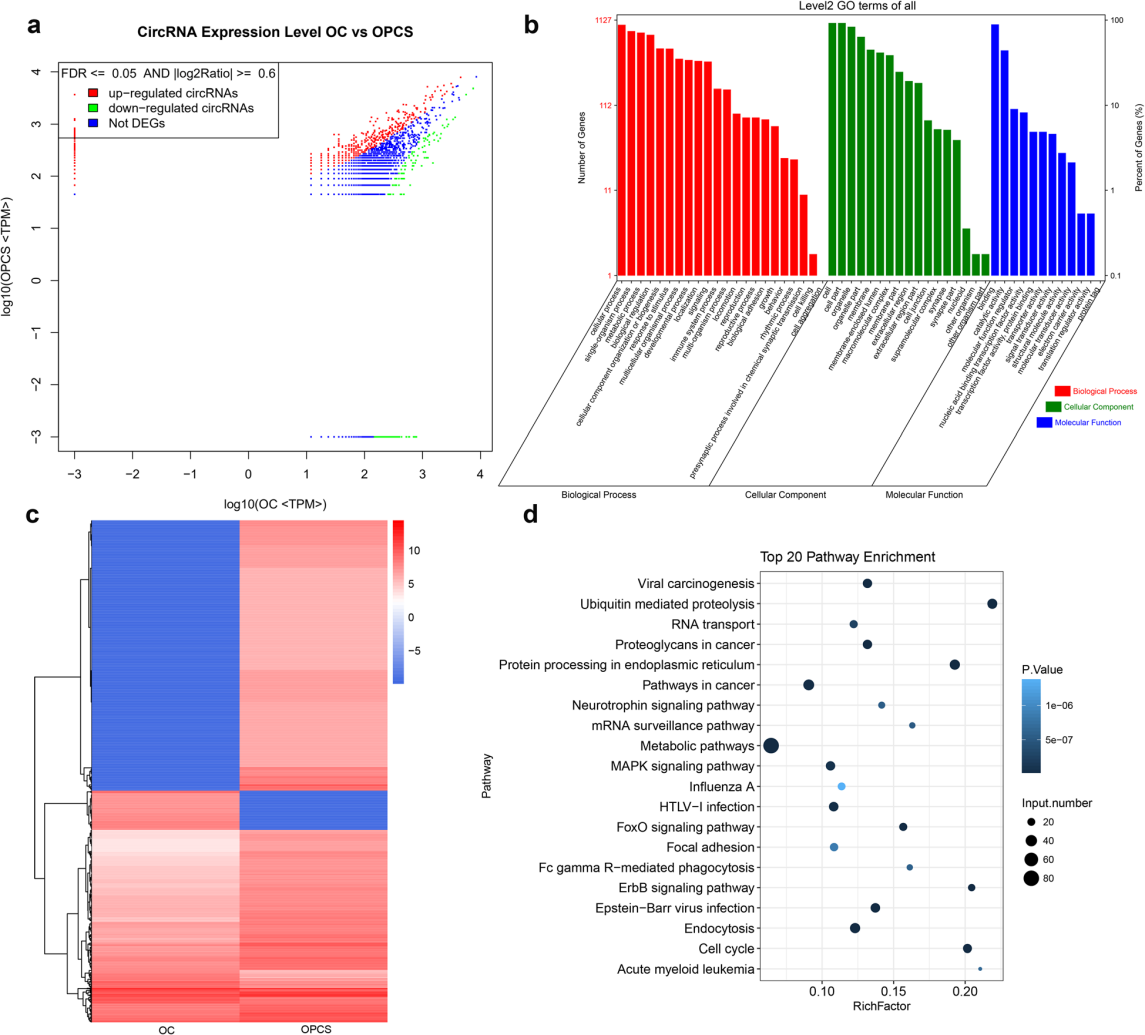
Differentially expressed circRNAs and their functions in the OC group and OPCS group. **a** Differentially expressed circRNAs in the OC group and OPCS group. **b** GO analysis of differentially expressed circRNAs. All GO items could be divided into three main subgroups: cellular components, molecular functions, and biological processes. **c** Heatmap analysis of the differentially expressed circRNAs in the OC and OPCS groups. **d** KEGG analysis of the top 20 differentially expressed circRNAs in the OC and OPCS groups. OCs, osteoclasts; OPCSs, osteogenic precursor cells

We identified the circRNAs that were differentially expressed in the OPCS and OC groups. Figure 3a shows that the number of upregulated DECs (1394) was higher than the number of downregulated DECs (214) in the OPCS group vs. the OC group. GO analysis revealed that the functions of these DECs were mainly distributed into three categories: cellular component, molecular function, and biological process (Fig. 3b). In the cellular component category, “cell,” “cell part,” and “organelle” were the three most enriched items. In the molecular function category, “binding” was the most enriched item. Finally, in the biological process category, “cellular process” and “single-organism process” were the most enriched items. We also produced a heat map to show the cluster relationships among these DECs (Fig. 3c). KEGG analysis of the top 20 DECs showed that enrichment was mostly in metabolic pathways and in the mitogen-activated protein kinase (MAPK) signaling pathway (Fig. 3d and Table 3).

**Table 3:** Top 20 differential expression circRNA list between the OC group and OPCS group

CircBase_ID	Gene ID	OC-TPM	OPCS-TPM	Log_**2**_ ratio (OPCS/OC)	Up-downregulation	***P*** value	FDR
hsa_circ_0003307	TALDO1	11.919	269.645	4.5	Up	2.05E− 07	1.77E− 05
hsa_circ_0007761	ATXN7	29.796	629.171	4.4	Up	3.76E− 15	1.14E− 12
hsa_circ_0076742	MCM3	35.756	629.171	4.137	Up	1.75E− 14	5.21E− 12
hsa_circ_0072202	C5orf42	11.919	202.234	4.085	Up	1.42E− 05	0.000626866
hsa_circ_0040123	PDXDC2P	17.878	269.645	3.915	Up	8.83E− 07	6.07E− 05
hsa_circ_0003260	RAP1B	17.878	269.645	3.915	Up	8.83E− 07	6.05E− 05
hsa_circ_0030213	LRCH1	11.919	179.763	3.915	Up	5.66E− 05	0.001799064
hsa_circ_0017065	B3GALNT2	11.919	179.763	3.915	Up	5.66E− 05	0.001795578
hsa_circ_0008842	ZNF367	23.837	337.056	3.822	Up	5.60E− 08	5.45E− 06
hsa_circ_0008883	CAPN15	11.919	157.293	3.722	Up	0.000222228	0.005249134
hsa_circ_0005585	NNT	476.741	44.941	− 3.407	Down	1.74E− 06	0.000102458
hsa_circ_0017077	LYST	435.026	44.941	− 3.275	Down	7.63E− 06	0.000353697
hsa_circ_0001541	ANKHD1	417.148	44.941	− 3.214	Down	1.43E− 05	0.000631106
hsa_circ_0001613	SENP6	613.804	67.411	− 3.187	Down	9.68E− 08	8.75E− 06
hsa_circ_0001346	RNF13	1501.734	179.763	− 3.062	Down	5.76E− 17	2.48E− 14
hsa_circ_0002394	SNX27	309.882	44.941	− 2.786	Down	0.000577398	0.012403449
hsa_circ_0046999	CEP192	280.085	44.941	− 2.64	Down	0.00156603	0.024002196
hsa_circ_0000099	AMY2B	280.085	44.941	− 2.64	Down	0.00156603	0.023979743
hsa_circ_0001432	MANBA	965.401	157.293	− 2.618	Down	6.75E− 10	8.84E− 08
hsa_circ_0001508	MSH3	274.126	44.941	− 2.609	Down	0.001908118	0.028164097

### DECs in an OC group vs. OC + AL group comparison

**Fig. 4:**
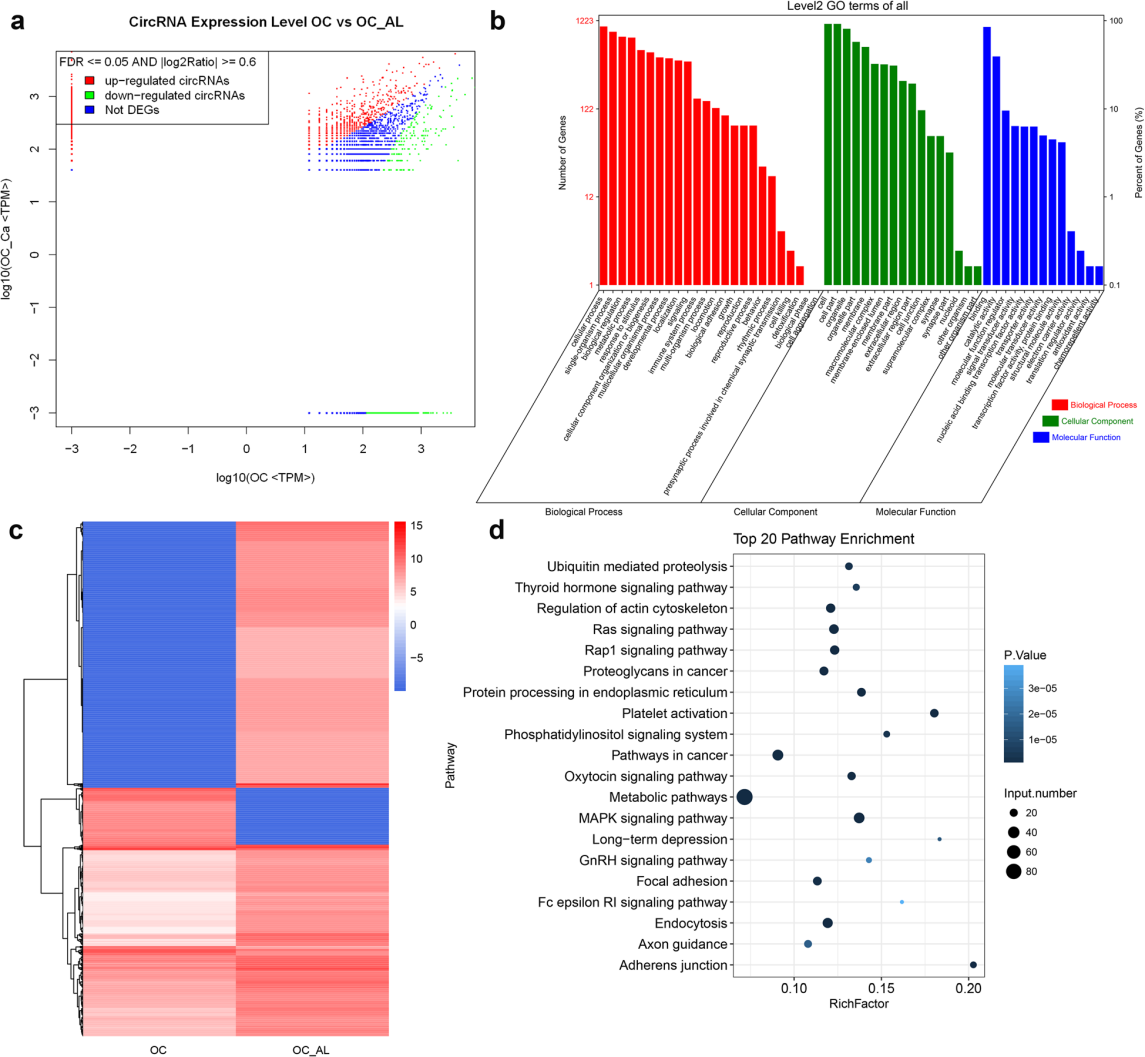
Differentially expressed circRNAs and their functions in the OC group and OC + AL group. **a** Differentially expressed circRNAs in the OC group and OC + AL group. **b** GO analysis of differentially expressed circRNAs. All GO items could be divided into three main subgroups: cellular components, molecular functions, and biological processes. **c** Heatmap analysis of the differentially expressed circRNAs in the OC and OC + AL groups. **d** KEGG analysis of the top 20 differentially expressed circRNAs in the OC and OC + AL groups. AL, alendronate; OCs, osteoclasts

We also identified the circRNAs that were differentially expressed in the OC and OC + AL groups. Figure 4a shows that the number of upregulated DECs (1434) was higher than the number of downregulated DECs (219) in this OC group vs. OPCS group comparison. As with the previous GO analysis, the functions of these DECs could be largely separated into the three categories cellular component, molecular function, and biological process (Fig. 4b). The most enriched items in the cellular component (“cell,” “cell part,” and “organelle”), molecular function (“binding”), and biological process (“cellular process” and “single-organism process”) categories were respectively identical to those in the OPCS group vs. OC group comparison. The heat map in Fig. 4c shows the cluster relationships among these DECs (Fig. 4c). As with the OPCS group vs. OC group comparison, KEGG analysis of the top 20 DECs showed that enrichment was mostly in metabolic pathways and in the MAPK signaling pathway (Fig. 4d and Table 4).

**Table 4:** Top 20 differential expression circRNA list between OC group and OC + AL group

CircBase_ID	Gene ID	OC-TPM	OC_AL-TPM	Log_**2**_ ratio (OC_AL/OC)	Up-downregulation	***P*** value	FDR
hsa_circ_0033144	BCL11B	11.919	2248.509	7.56	Up	9.34E− 43	1.14E− 39
hsa_circ_0009125	HABP4	35.756	2981.719	6.382	Up	1.92E− 52	3.05E− 49
hsa_circ_0083766	EPHX2	11.919	830.971	6.123	Up	1.37E− 15	2.14E− 13
hsa_circ_0007426	FNBP1	11.919	782.09	6.036	Up	1.13E− 14	1.53E− 12
hsa_circ_0005035	IGF1R	17.878	977.613	5.773	Up	1.67E− 17	3.15E− 15
hsa_circ_0063809	CELSR1	17.878	830.971	5.539	Up	8.57E− 15	1.19E− 12
hsa_circ_0005660	NFIX	11.919	488.806	5.358	Up	3.16E− 09	1.94E− 07
hsa_circ_0033476	MARK3	11.919	488.806	5.358	Up	3.16E− 09	1.93E− 07
hsa_circ_0003489	CDK8	11.919	488.806	5.358	Up	3.16E− 09	1.92E− 07
hsa_circ_0001730	EPHB4	53.633	2150.748	5.326	Up	1.63E− 34	1.23E− 31
hsa_circ_0000566	VRK1	1853.331	97.761	− 4.245	Down	3.16E− 13	3.86E− 11
hsa_circ_0000826	ANKRD12	3629.191	244.403	− 3.892	Down	7.83E− 24	2.53E− 21
hsa_circ_0005615	NFATC3	3259.717	244.403	− 3.737	Down	5.80E− 21	1.59E− 18
hsa_circ_0004658	EMILIN2	1251.445	97.761	− 3.678	Down	1.66E− 08	8.44E− 07
hsa_circ_0000284	HIPK3	23306.676	1906.345	− 3.612	Down	1.52E− 139	6.05E− 136
hsa_circ_0086735	UBAP2	2407.542	244.403	− 3.3	Down	1.85E− 14	2.44E− 12
hsa_circ_0000994	SLC8A1	7472.915	879.851	− 3.086	Down	2.15E− 40	2.13E− 37
hsa_circ_0002538	KLHL8	709.152	97.761	− 2.859	Down	0.000201768	0.003278317
hsa_circ_0006156	FNDC3B	4058.258	635.448	− 2.675	Down	7.46E− 20	1.77E− 17
hsa_circ_0006595	ST3GAL3	1489.816	244.403	− 2.608	Down	9.13E− 08	3.79E− 06

### Network of circRNA–miRNA–mRNA

**Fig. 5:**
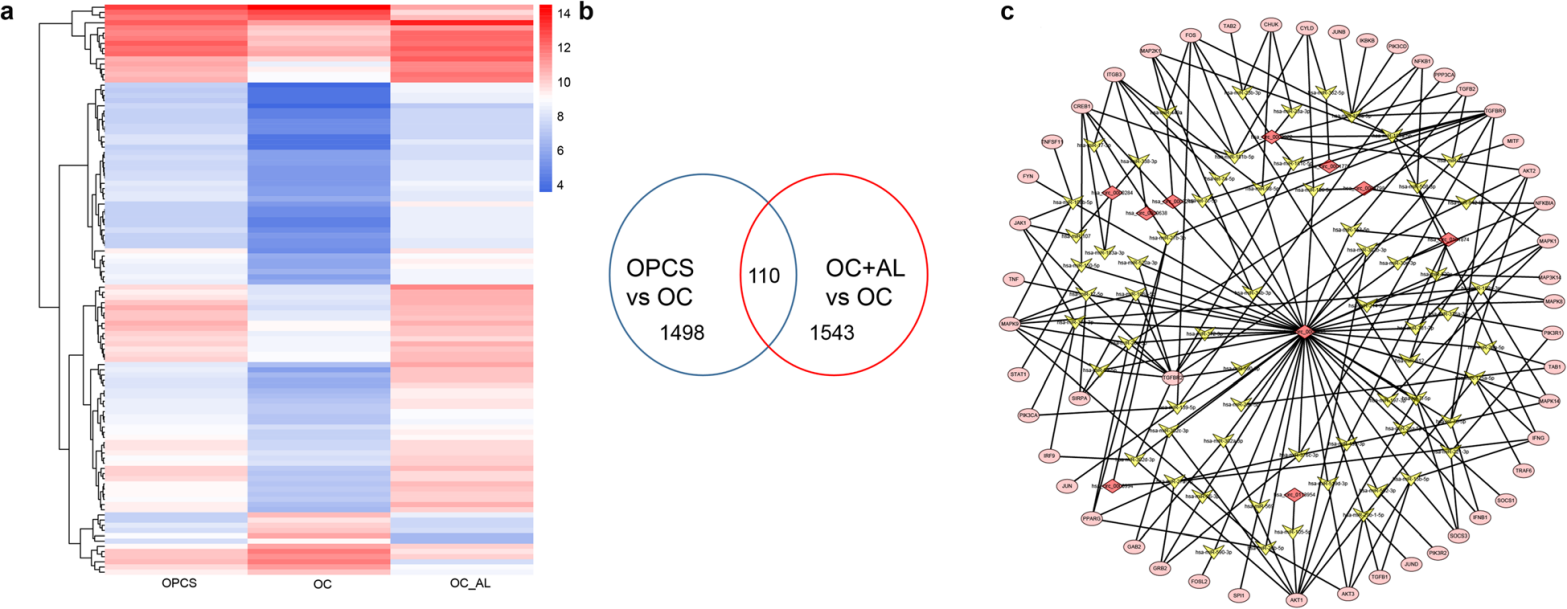
Differentially expressed circRNAs and their regulation networks using the top 20 differentially expressed circRNAs in the OC, OPCS, and OC + AL groups. **a** Heatmap analysis of the differentially expressed circRNAs among the three treatment groups. **b** Venn diagram showing the relationships between the differentially expressed circRNAs among the three treatment groups. **c** circRNA–miRNA–mRNA regulation network analysis of the top 20 differentially expressed circRNAs among the three treatment groups

**Fig. 6:**
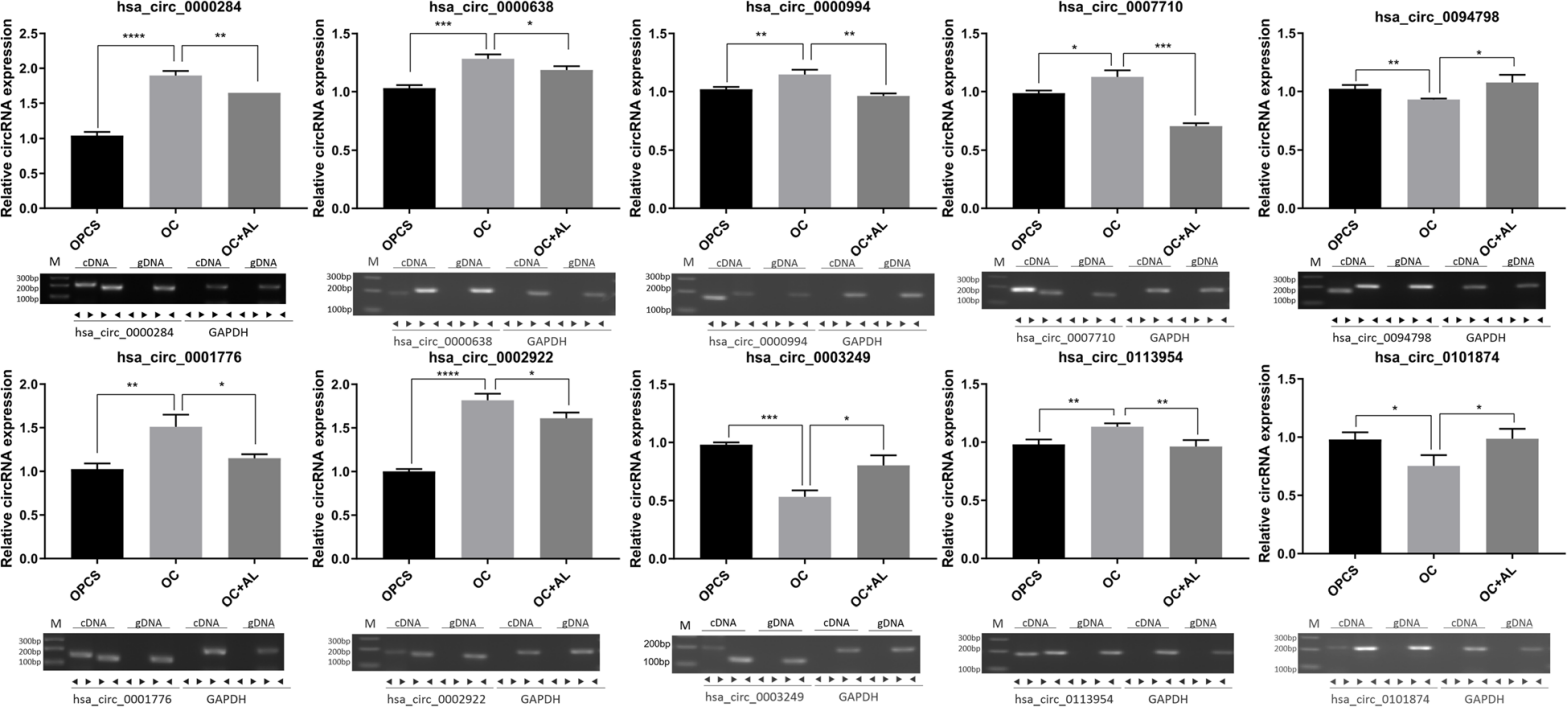
qPCR analysis and agarose gel electrophoresis analysis of 10 differentially expressed circRNAs selected from the circRNA–miRNA–mRNA regulation network in the OPCS, OC, and OC + AL groups. *P < 0.05, **P < 0.01, ***P < 0.001, and ****P < 0.0001. GAPDH was used as an internal reference. AL, alendronate; OCs, osteoclasts; OPCSs, osteogenic precursor cells

To further examine the function of DECs, we screened the overlap of DECs among the three treatment groups: 110 circRNAs were differentially expressed among these groups. Of the 110 DECs, 95 and 15 were upregulated and downregulated after AL treatment, respectively (Fig. 5a, b). In addition, we constructed a circRNA–miRNA–mRNA network. The circRNAs related to OC differentiation were selected using the following criteria: (1) circRNAs were sourced from exons, and (2) the length of circRNAs ranged from 300 to 1200 bp. Figure 5 shows that hsa_circ_0002922 bound to hsa-miR-181b-5p to regulate the expression of MAP2K1 and hsa_circ_0007710 bound to hsa-miR-197-3p to regulate the expression of MAPK1, and it also bound to hsa-miR-20a-5p to regulate the expression of MAPK9. The 20 top DECs are shown in Table 5. Figure 5c shows that the top 10 DECs played key roles in the circRNA–miRNA–mRNA network. Furthermore, the expression levels of 10 DECs in the key networks (hsa_circ_0000284, hsa_circ_0000638, hsa_circ_0000994, hsa_circ_0001776, hsa_circ_0002922, hsa_circ_0003249, hsa_circ_0007710, hsa_circ_0094789, hsa_circ_0113954, and hsa_circ_0101874) were also validated in the three treatment groups using qPCR (Fig. 6). The expression levels of hsa_circ_0000284, hsa_circ_0000638, hsa_circ_0000994, hsa_circ_0001776, hsa_circ_0002922**,** hsa_circ_0007710, and hsa_circ_0113954 were significantly increased in the OC group compared with those in the OPCS group (*P* < 0.05). However, the expression levels of these DECs were significantly decreased in the OC + AL group compared with those in the OC group (*P* < 0.05). Furthermore, the expression levels of hsa_circ_0003249, hsa_circ_0094789, and hsa_circ_0101874 were significantly decreased in the OC group compared with those in the OPCS group (*P* < 0.05). In contrast, the expression levels of these DECs were significantly increased in the OC + AL group compared with those in the OC group (*P* < 0.05). Finally, we confirmed the identity of the top 10 DECs via agarose gel electrophoresis (Fig. 6).

**Table 5:** Top 20 differential expression circRNA list between three groups

CircBase_ID	Gene ID	OC-TPM	OC_AL-TPM	OPCS-TPM	Log_**2**_ ratio (OPCS/OC)	Up-downregulation (OPCS/OC)	***P*** value	FDR	Log_**2**_ ratio (OC_AL/OC)	Up-downregulation (OC_AL/OC)	***P*** value	FDR
hsa_circ_0008883	CAPN15	11.919	439.926	157.293	3.722	Up	0.000222228	0.005249134	5.206	Up	2.46E− 08	1.24E− 06
hsa_circ_0007761	ATXN7	29.796	782.09	629.171	4.4	Up	3.76E− 15	1.14E− 12	4.714	Up	1.26E− 12	1.38E− 10
hsa_circ_0000053	STK40	17.878	439.926	134.822	2.915	Up	0.00232928	0.033387027	4.621	Up	9.62E− 08	3.97E− 06
hsa_circ_0004137	RBM23	17.878	342.164	157.293	3.137	Up	0.000663894	0.014076789	4.258	Up	4.73E− 06	0.000117454
hsa_circ_0042079	GAS7	17.878	342.164	134.822	2.915	Up	0.00232928	0.033125964	4.258	Up	4.73E− 06	0.00011727
hsa_circ_0001030	EXOC6B	101.307	1857.464	292.115	1.528	Up	0.00431496	0.043788952	4.197	Up	7.48E− 25	2.96E− 22
hsa_circ_0051849	SNRNP70	71.511	1173.135	247.174	1.789	Up	0.00302166	0.041494591	4.036	Up	7.88E− 16	1.26E− 13
hsa_circ_0002161	GSPT1	41.715	635.448	179.763	2.107	Up	0.00418748	0.042733703	3.929	Up	3.53E− 09	2.13E− 07
hsa_circ_0071099	ARHGAP10	113.226	1661.942	696.582	2.621	Up	2.52E− 10	3.59E− 08	3.876	Up	8.09E− 21	2.17E− 18
hsa_circ_0008156	ITPA	23.837	342.164	157.293	2.722	Up	0.001623886	0.024749898	3.843	Up	1.28E− 05	0.000297513
hsa_circ_0000566	VRK1	1853.331	97.761	471.878	− 1.974	Down	1.92E− 13	4.63E− 11	− 4.245	Down	3.16E− 13	3.86E− 11
hsa_circ_0000826	ANKRD12	3629.191	244.403	1348.224	− 1.429	Down	8.47E− 17	3.55E− 14	− 3.892	Down	7.83E− 24	2.53E− 21
hsa_circ_0000284	HIPK3	23306.676	1906.345	9122.98	− 1.353	Down	2.00E− 92	1.64E− 88	− 3.612	Down	1.52E− 139	6.05E− 136
hsa_circ_0000994	SLC8A1	7472.915	879.851	4808.665	− 0.636	Down	4.27E− 10	6.03E− 08	− 3.086	Down	2.15E− 40	2.13E− 37
hsa_circ_0003692	FNDC3B	1120.341	195.523	292.115	− 1.939	Down	2.00E− 08	2.06E− 06	− 2.519	Down	6.75E− 06	0.000163499
hsa_circ_0007710	ELF2	1191.852	244.403	224.704	− 2.407	Down	4.54E− 11	7.22E− 09	− 2.286	Down	1.00E− 05	0.000240639
hsa_circ_0002922	ZNF124	2222.805	488.806	943.757	− 1.236	Down	5.61E− 09	6.25E− 07	− 2.185	Down	2.06E− 09	1.28E− 07
hsa_circ_0001346	RNF13	1501.734	342.164	179.763	− 3.062	Down	5.76E− 17	2.48E− 14	− 2.134	Down	1.54E− 06	4.85E− 05
hsa_circ_0000119	MAN1A2	3355.065	782.09	1235.872	− 1.441	Down	8.35E− 16	2.91E− 13	− 2.101	Down	4.14E− 13	4.94E− 11
hsa_circ_0000638	ETFA	744.908	195.523	179.763	− 2.051	Down	2.70E− 06	0.000154121	− 1.93	Down	0.00211178	0.024373077

## Discussion

OCs are the main functional cells involved in bone resorption during bone remodeling. Their differentiation and activity are regulated by a variety of hormones and cytokines, including MCSF and RANKL: these two cytokines are critical for the regulation of OC-like cell differentiation in vitro [20]. Therefore, we selected both MCSF and RANKL to induce THP-1 cells into OCs and showed that they were functionally able to induce OC differentiation, which is a finding consistent with that of a previous study [21].

The anti-bone resorption effects of AL are mainly achieved via the inhibition of OCs. AL binds to non-hydrolyzable ATP analogs on the surface of OC membranes and inhibits the action of ATP-dependent intracellular enzymes, which in turn significantly inhibits the effects of OCs [22]; specifically, OCs lose the ability to absorb bone, which affects bone resorption and bone turnover rate [23, 24]. Bisphosphonates such as AL are currently the most commonly used drugs for the treatment of OP; they can specifically bind to hydroxyapatite in the bone so are strong inhibitors of bone resorption but have little effect on other tissues [25]. Srisubut et al. found that AL promotes bone formation in bioactive glass grafting in rats [26], and Jensen et al. demonstrated that orally administering AL to dogs with prosthesis implantations promotes bone formation around a good prosthesis [27]. These studies indicate that AL plays a role in promoting local bone formation; similarly, we found that AL effectively inhibits OC differentiation.

We also found that 1394 and 214 circRNAs were differentially upregulated and downregulated, respectively, in the OPCS and OC groups, whereas 1434 and 219 circRNAs were differentially upregulated and downregulated, respectively, in the OC and OC + AL groups. Notably, GO and KEGG analysis revealed that gene functions and signaling pathways were similarly enriched between these two groups of DECs. This indicates that the same genes might participate in both OC differentiation and inhibition. For example, the MAPK signaling pathway was enriched in both sets of DECs. The MAPK pathway is important in the signaling network of eukaryotic cells: it transduces extracellular mitosis signals to the cell nuclei [28]. Activated MAPK regulates a variety of cellular physiological processes such as growth, division, differentiation, and apoptosis through phosphorylation and ubiquitination [29]. Previous studies have shown that the MAPK pathway regulates the osteoprotegerin/RANKL intracellular pathway involved in the balance of bone metabolism.

The MAPKs associated with OC differentiation mainly comprise three subfamilies: extracellular signal-regulated protein kinase (ERK), p38MAPK, and c-Jun amino-terminal kinase [30]. The MAPK pathway is also considered to be an important pathway in inflammatory bone injury [31]. Other studies have shown that the p38 inhibitor SB203580 and the ERK inhibitor U0126 significantly reduce the activity of TRAP during OC formation and thereby inhibit the formation and function of OCs [32]. In addition, the circRNA CDR1as can inhibit the expression of miR-7, which releases the negative regulation of miR-7 on growth differentiation factor 5, further activates the p38MAPK pathway and pSmad1/5/8, and promotes osteogenesis of periodontal ligament stem cells [33]. These results are consistent with our finding that circRNAs might bind to miRNA to regulate the MAPK pathway during differentiation of OCs treated with AL.

To date, there is a lack of research on circRNAs in the skeletal system. Some early studies have reported the expression of long non-coding RNAs during chondrocyte differentiation and osteoblast differentiation [34]; however, the expression and function of circRNAs in the process of OC formation had previously not been demonstrated. Thus, our study is the first to report the expression profiles of circRNAs during the differentiation of OCs treated with AL.

## Conclusions

In summary, here, we successfully induced OPCSs to differentiate into OCs, showed that AL could suppress OPCSs from differentiating into OCs, and identified 10 DECs that were involved in the regulation of this process. These 10 circRNAs might be important to the treatment of OP; this speculation requires further enquiry.

## Data Availability

Any information used and analyzed during this study is available from the corresponding author on reasonable request.

## Abbreviations

OP: Osteoporosis
OC: Osteoclast
circRNAs: Circular RNAs
AL: Alendronate
IL: Interleukin
MCSF: Macrophage colony-stimulating factor
RANKL: Receptor activator of nuclear factor-κB ligand
PMA: Phorbol-12 myristate-13 acetate
OPCS: Osteoclast precursor
TRAP: Tartrate-resistant acid phosphatase
CTSK: Cathepsin K
DEC: Differentially expressed circRNA
MAPK: Mitogen-activated protein kinase
ERK: Extracellular signal-regulated protein kinase
